# Surface lattice resonances and magneto-optical response in magnetic nanoparticle arrays

**DOI:** 10.1038/ncomms8072

**Published:** 2015-05-07

**Authors:** M. Kataja, T. K. Hakala, A. Julku, M. J. Huttunen, S. van Dijken, P. Törmä

**Affiliations:** 1 NanoSpin, Department of Applied Physics, Aalto University School of Science, FI-00076 Aalto, Finland; 2 COMP Centre of Excellence, Department of Applied Physics, Aalto University, FI-00076 Aalto, Finland

## Abstract

Structuring metallic and magnetic materials on subwavelength scales allows for extreme confinement and a versatile design of electromagnetic field modes. This may be used, for example, to enhance magneto-optical responses, to control plasmonic systems using a magnetic field, or to tailor magneto-optical properties of individual nanostructures. Here we show that periodic rectangular arrays of magnetic nanoparticles display surface plasmon modes in which the two directions of the lattice are coupled by the magnetic field-controllable spin–orbit coupling in the nanoparticles. When breaking the symmetry of the lattice, we find that the optical response shows Fano-type surface lattice resonances whose frequency is determined by the periodicity orthogonal to the polarization of the incident field. In striking contrast, the magneto-optical Kerr response is controlled by the period in the parallel direction. The spectral separation of the response for longitudinal and orthogonal excitations provides versatile tuning of narrow and intense magneto-optical resonances.

## Abstract (web summary)

By incorporating magnetic materials into periodic nanostructures, additional control over the magneto-optical response of the system can be introduced. Kataja *et al*. show that arrays of magnetic nanoparticles exhibit Fano-type surface plasmon resonances with cross-coupling tuned by the lattice symmetry.

## Graphical abstract

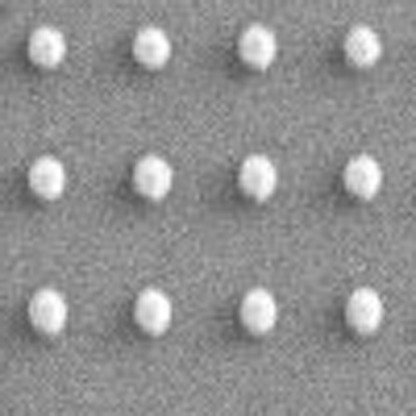

The ability to tune the optical response of magnetic materials via external magnetic fields has initiated the interest to use magnetic materials in plasmonics to implement active tunability into plasmonic structures^1^,^2^. Further, combining plasmonics effects with magnetic materials has been shown to enhance the magneto-optical response^3^,^4^,^5^,^6^,^7^,^8^,^9^. While in bulk materials the magneto-optical activity is governed by the spin–orbit interaction, a property that is intrinsic to a given material, it has recently been shown that the magneto-optical response of magnetic nanoparticles is governed by localized surface plasmon resonances (LSPRs)^10^,^11^. Further studies with elliptical particles revealed that the LSPR-polarized orthogonally with respect to the driving field and light propagation direction was solely responsible for the response^12^,^13^. The system was accurately described by two orthogonal, damped harmonic oscillators corresponding to a light-induced longitudinal LSPR-polarized parallel to the incident field (the first oscillator) and a spin–orbit-induced transverse LSPR-polarized perpendicular to the driving field (the second oscillator). In contrast to vastly studied plasmon resonances of non-magnetic metallic nanoparticles in which a simple relation exists between the driving and the emitted field, magnetic nanoparticles exhibit a richer and magnetic field tunable plasmonic phase and amplitude response due to coupling between two orthogonal oscillators.

Here we address a question that naturally arises from the coupling between two orthogonal directions—what happens when the magnetic nanoparticles are arranged in a two-dimensional rectangular lattice? It is known that periodic arrangement of metallic nanoparticles (or holes) may lead to Fano-type surface lattice resonances (SLRs) with extremely narrow and asymmetric line shapes^14^,^15^,^16^,^17^,^18^,^19^,^20^. Such systems have recently shown to exhibit strong coupling with molecules^21^,^22^,^23^. However, no specific effects were associated with the two-dimensional nature of the lattice, that is, the response was insensitive to the periodicity parallel to the polarization of the driving field; in this case, the relevant lattice modes were so-called perpendicular SLRs, which can be excited with TE-polarized light and are prominent in symmetric refractive index environments. Recently, however, a transverse magnetic-like parallel SLR mode was identified in two-dimensional arrays of gold nanoparticles^24^,^25^; the system response was sensitive to the periodicity parallel to the polarization of the incident light and insensitive to the perpendicular periodicity due to a large difference in periodicities^25^ or due to an inhomogeneous refractive index environment^24^. Furthermore, symmetric two-dimensional arrays of gold nanoparticles were studied with transverse magnetic-polarized light and a nonzero angle of incidence^26^, which resulted in lasing when embedded in a gain medium^27^.

Here we explore the interplay between periodic arrangements of magnetic nickel (Ni) nanoparticles and their single-particle optical and magneto-optical response. For the first time, we show that the LSPR supported by the Ni nanoparticles hybridizes with the narrow line width diffracted orders of the lattice (Rayleigh anomalies) via radiation fields. This results in a prominent Fano-type SLR with a very narrow asymmetric line shape. We show that the polar magneto-optical Kerr response is strongly modified by the presence of the SLR and that the magneto-optical activity is governed by the SLR mode that is associated with the periodicity parallel to the driving field.

## Results

### Effects induced by periodicity and magnetic field

**Figure 1:**
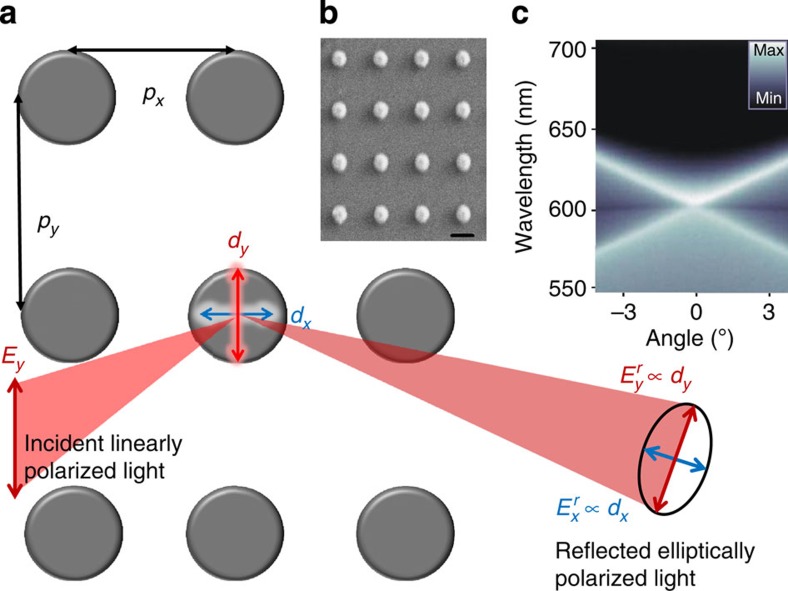
Magnetic nanoparticle arrays and experimental configuration. (**a**) Schematic of the system under study. For magnetic nanoparticle arrays, we show that, in the presence of magnetic material, the system response is governed not only by the induced dipole moment *d*_*y*_ parallel to the driving field *E*_*y*_ and the lattice period *p*_*x*_ (direction of dipole radiation), but also by the spin–orbit-induced and magnetic-field tunable dipole moment *d*_*x*_ and lattice period *p*_*y*_. (**b**) Scanning electron micrograph of an ordered rectangular array of cylindrical Ni nanoparticles. Scale bar, 200 nm. (**c**) Angle- and wavelength-resolved optical transmission of a sample with *p*_*x*_=*p*_*y*_=400 nm and with particle diameter 120 nm, showing crossing of the <+1, 0> and <−1, 0> diffracted orders of the lattice at normal incidence.

In Fig. 1a, we show the schematic of the system under study. A two-dimensional array of metallic nanoparticles with periodicities *p*_*x*_ and *p*_*y*_ are driven by the external electric field *E*_*y*_, which induces a dipole moment *d*_*y*_ and radiation predominantly along the *x* axis in each nanoparticle. An important special case arises when the angle of incidence is zero and the particle spacing *p*_*x*_=*λ*/*n*, where *λ* is the free space wavelength of the driving field and *n* is the refractive index of the homogeneous medium surrounding the particles. Under these conditions, radiation fields from all the particles interfere constructively at each particle location. In the absence of magnetization, the system response is governed by *d*_*y*_ and *p*_*x*_. If, however, the particles are composed of a magnetic material with out-of-plane magnetization, then *E*_*y*_, due to spin–orbit coupling, induces an additional dipole moment *d*_*x*_. As a result, the polarization of the reflected light rotates and becomes elliptical. Thus, the system response may be influenced not only by *d*_*y*_ and *p*_*x*_, but also by *d*_*x*_ and *p*_*y*_. Whether and how *d*_*x*_ and *p*_*y*_ affect the response, however, is an open question and thus motivates the present study.

### Optical and magneto-optical response

To study the optical and magneto-optical response of the samples, we measured the normal incidence optical reflectivity and polar magneto-optical Kerr ellipticity *ɛ* and rotation *θ* of the reflected light field with two incident light polarizations *E*_*x*_ and *E*_*y*_. Since measurements were performed in reflection geometry at normal incidence, the reflected electric field strengths are directly proportional to the amplitudes of the induced dipoles so that *E*^r^_*y*_/*E*^r^_*x*_=*d*_*y*_/*d*_*x*_ (see also Fig. 1a). Thus, we define the Kerr ellipticity *ɛ*=Im(*d*_*y*_/*d*_*x*_) and rotation *θ*= Re(*d*_*y*_/*d*_*x*_) for *E*_*x*_ polarization of the incident field. Similarly, *ɛ*=Im(*d*_*x*_/*d*_*y*_) and *θ*=Re(*d*_*x*_/*d*_*y*_) for *E*_*y*_ polarization of the incident field. The spin–orbit-induced dipole moment depends on the direction of magnetization in the Ni nanoparticles, and hence it can be controlled by an external magnetic field. In the magneto-optical Kerr effect measurements, full hysteresis curves were recorded for each wavelength in out-of-plane magnetic fields up to±400 mT, which is sufficient to fully saturate the Kerr ellipticity and Kerr rotation signals. The experimental values of *ɛ* and *θ* were subsequently extracted from the data by averaging of the saturated magneto-optical Kerr responses. The results are compared with a numerical model based on discrete dipole approximation (DDA)^28^,^29^,^30^. The measurements were carried out for three different particle diameters—80, 120 and 160 nm (the results for 80 and 160 nm diameter particles are shown in Supplementary Figs 1 and 2). For a detailed description of the sample fabrication, magneto-optical measurements and the numerical model see the Methods section.

**Figure 2:**
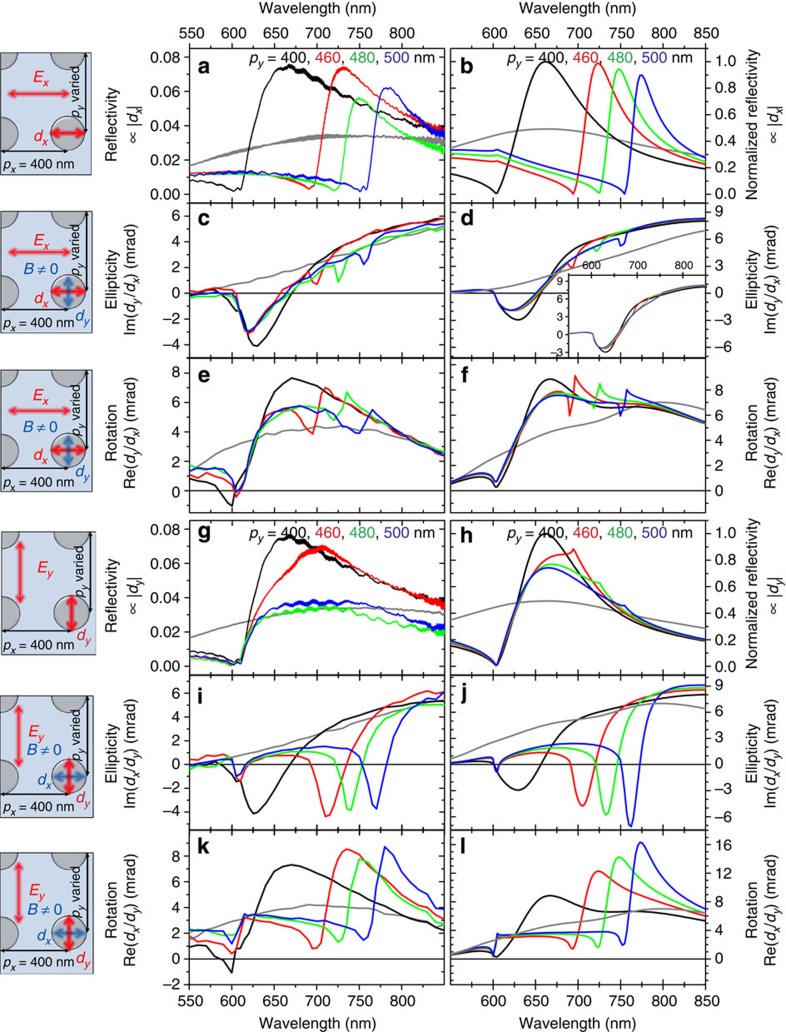
Optical and magneto-optical responses of the samples. The schematics on the left illustrate the direction of incident polarization and the induced dipole moments that are probed in the measurements. The graphs in the middle and right show experimental data and results from DDA simulations, respectively. The black, red, green and blue curves correspond to the periodicities *p*_*y*_=400, 460, 480 and 500 nm, in all the figures. The grey line corresponds to a random sample. (**a**,**b**) Normal incidence optical reflectivity for incident light polarization *E*_*x*_. The magneto-optical Kerr ellipticity (**c**,**d**) and rotation (**e**,**f**) with polarization *E*_*x*_. Normal incidence optical reflectivity (**g**,**h**), the magneto-optical Kerr ellipticity (**i**,**j**) and rotation (**k**,**l**) for polarization *E*_*y*_. Note that the DDA simulations were carried out with a small (0.04°) rotation of incident light polarization to account for the *p*_*y*_-dependent features in the experimental spectra (**c**,**e**). The inset of **d** shows a DDA result without this rotation. In contrast, the small features in **h** are not caused by a small sample tilt (see text for discussion).

Figure 2a shows the optical reflectivity of the samples composed of 120 nm diameter particles for linear polarized light along the *x* axis. The periodicities (*p*_*x*_ × *p*_*y*_) were: (1) 400 × 400 nm, (2) 400 × 460 nm, (3) 400 × 480 nm and (4) 400 × 500 nm. Also shown is the result for a random sample, where the particle size, number and orientation are the same as in the 400 × 400 nm sample, but the particles are randomly distributed along the sample. As no constructive interference effects are present for the randomly distributed particles (grey curve), the response is dominated by a broad LSPR centred at 750 nm. For the 400 × 400 nm sample, a radically different, strongly asymmetric and wavelength-dependent response is obtained as compared with the random sample. The reflectivity minimum is located at 600 nm, which corresponds to the <+1, 0> and <−1, 0> diffracted orders of the lattice further confirmed by angle-resolved transmission measurements (see Fig. 1c). This response is due to radiative coupling of the particles in a periodic array, hybridizing the broad single-particle resonance with the narrow delocalized diffracted orders of the lattice, resulting in a prominent Fano line shape (see also Supplementary Figs 3–6). Similar Fano-type resonances have been previously identified for noble metal nanoparticles in periodic arrangements by our and other groups^15^,^18^,^20^,^21^, for periodic corrugations in magnetic films^31^,^32^,^33^ and for individual dimer structures composed of noble- and magnetic metals^34^,^35^, but to our knowledge this is the first study in which SLRs are reported for magnetic Ni nanoparticle arrays. As the high ohmic losses in Ni result in very broad LSPR (*Q* factor ∼3 in the present case), radiative coupling of such lossy resonators is not obvious. Nevertheless, the coupling is evident from the asymmetric line shapes and the angle-resolved measurements.

For the asymmetric samples, the resonance is redshifted to 700, 725 and 750 nm as the sample periodicity *p*_*y*_ is increased to 460, 480 and 500 nm, respectively (see red, green and blue curves in Fig. 2a). This is due to the gradual redshift of the constructive interference condition of the radiation fields along the *y* axis of the array. All the reflectivity curves are accurately reproduced by the numerical DDA method, see Fig. 2b.

The measured Kerr ellipticity and rotation for an incident polarization along the *x* axis are shown in Fig. 2c,e, respectively. Both the ellipticity and rotation of the random sample (grey line) are mainly featureless and dominated by a broad LSPR. Contrary to the random sample, the 400 × 400 nm sample shows features in both ellipticity and rotation corresponding to the SLR at 600 nm (see the black lines in Fig. 2c,e). We next consider samples in which the periodicity *p*_*x*_ is maintained at 400 nm while *p*_*y*_ is gradually increased to break the symmetry of the lattice. All these samples exhibit virtually identical magneto-optical responses around 600 nm, independent of *p*_*y*_. This behaviour is very similar to the purely optical response with opposite polarization *E*_*y*_, where the most prominent features are also at 600 nm (see the red, green and blue curves in Fig. 2g). The magneto-optical results are in good agreement with the DDA calculations (see Fig. 2d,f). We note that the *p*_*y*_-dependent features at (700, 725 and 750 nm) in the experimental data are due to a small sample tilt and that they are reproduced by DDA if a sample tilt of about 0.04° is assumed with respect to the incident field polarization. The inset of Fig. 2d shows the DDA spectra in the absence of the tilt. The emergence of these *p*_*y*_-dependent features with increasing rotation of sample axis is also evident in Supplementary Fig. 7.

Let us now inspect in detail the optical responses for linear polarized light along the *y* axis. Three issues are evident from Fig. 2g: (1) the optical reflectivity of the random sample shows only LSPR-dominated response, (2) the *p*_*x*_=400 nm, *p*_*y*_=400 nm sample shows strong features at 600 nm and (3) due to lack of asymmetry, the two aforementioned samples show polarization-independent reflectivities (compare the gray and black curves in Fig. 2a,g). Furthermore, apart from the intensity, the optical responses of all the asymmetric samples (*p*_*y*_ ≠ 400 nm) are very similar to the 400 × 400 nm sample (compare the black to red, green and blue curves in Fig. 2g). This is because the principal radiation direction of each particle is along the short period (*p*_*x*_=400 nm), so the constructive interference condition for the radiation is met at the same wavelength as for the 400 nm × 400 nm sample. This is further confirmed by the DDA model (see Fig. 2h). The small *p*_*y*_-dependent features at 700, 725 and 750 nm in the optical reflection spectra (see the red, green and blue curves in Fig. 2h) are likely due to a parallel SLR mode, as recently reported in the context of plasmonic nanoparticle arrays^24^,^25^. In contrast to refs 24, 25, however, our system is studied in a symmetric dielectric environment under normal incidence. Under these conditions, coupling to parallel SLRs is weak, and consequently, this mode is virtually undetectable in the experimental reflectivity curves (Fig. 2g).

The Kerr ellipticity and rotation for random and symmetric 400 × 400 nm samples with incident *E*_*y*_ polarization are, as they should be, similar to the data for *E*_*x*_ polarization due to symmetry (to compare, see the grey and black curves for ellipticity in Fig 2i,c and for rotation in Fig. 2k,e). However, the Kerr ellipticity and rotation of the asymmetric samples show strong features (minima) at gradually increasing wavelengths (700, 725 and 750 nm) as a function of increasing periodicity *p*_*y*_ (460, 480 and 500 nm), see the red, green and blue curves in Fig. 2i,k. Notably, the features appear at the same wavelengths as the optical reflection minima for orthogonal (*E*_*x*_) polarization (see Fig. 2a), and are in good agreement with the numerical DDA model. The small features at 600 nm, again, are present in the DDA calculations only if a slight sample tilt of 0.04° is introduced with respect to the driving field. We also carried out similar measurements for arrays composed of 80 and 160 nm diameter particles (see Supplementary Figs 1 and 2). Increasing optical reflectivity is observed with larger particles. Owing to a gradual redshift of LSPR for increasing particle size, slightly different shapes are obtained for the reflectivity, Kerr rotation and Kerr ellipticity curves, but otherwise the results are qualitatively similar to the ones shown for 120 nm particles in Fig. 2. The measurement results are again in good agreement with the corresponding DDA calculations.

Our results can be summarized as follows. While the optical response of the samples is governed by the lattice periodicity perpendicular to the polarization of the driving field, the magneto-optical response is dominated by the periodicity parallel to the polarization. The SLRs produce strong and spectrally sharp features in the magneto-optical response, which can be accurately controlled by tuning the lattice periodicity.

### Analytical model

**Figure 3:**
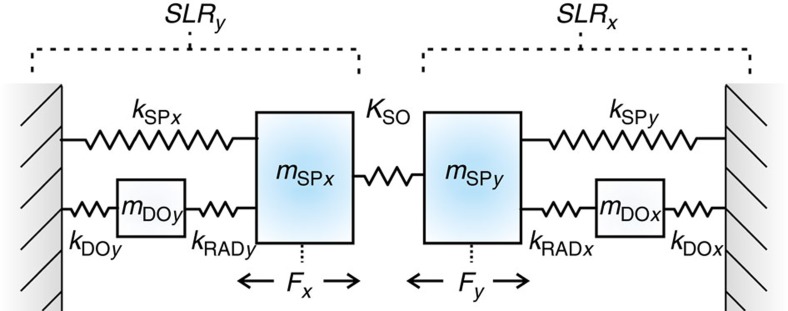
Oscillator model for optical and magneto-optical response of magnetic nanoparticle arrays. The single-particle LSPR in the *x* (*y*) direction is composed of *m*_SP*x*(*y*)_, *k*_SP*x*(*y*)_ and the damping term *γ*_SP*x*(*y*)_. The diffracted orders of the array are described by (*m*, *k*, *γ*)_DO*y*(*x*)_, the radiative coupling between the LSPRs and the diffracted orders by *k*_RAD*y*(*x*)_, and the spin–orbit coupling between the two orthogonal oscillators inside the particles by *K*_SO_. We chose our model parameters to mimic the *p*_*x*_=400 nm, *p*_*y*_=500 nm sample: in this case the SLR_*x*_ resonance is at 600 nm while the SLR_*y*_ is at 750 nm.

To gain physical insight to the observed phenomenon beyond the DDA calculations, we developed a simple analytical model with four coupled oscillators (see Fig. 3). The subindices SP*x*(*y*) refer to the LSPR in each particle in the *x*(*y*)-direction (for our cylindrical nanoparticles they are the same) and DOx(y) refers to the periodicity-dependent diffracted orders in the lattice. The parameters *k*_DO_ and *m*_DO_ in the model are connected to the sample resonance frequencies and thus to periodicities *p* by the relation *k*_DO*x*(*y*)_/*m*_DO*x*(*y*)_=(2*πc*/(*np*_*x*(*y*)_))^2^, where *n* is the refractive index of the medium. The radiation-induced hybridization of LSPRs and diffracted order modes results in SLRs. This is implemented into the model by the coupling springs *k*_RAD*y*_ and *k*_RAD*x*_. The spin–orbit-induced coupling between SP_*x*_ and SP_*y*_ is described by *K*_SO_. The entire system thus consists of four masses and seven springs. The spring constants (*k*), masses (*m*) and damping (*γ*) of each oscillator are chosen to match the experimentally-measured resonance frequencies and line widths, see Supplementary Figs 3–6 and Supplementary Table 1. The incident field polarization *E*_*x*_ corresponds to the condition *F*_*x*_≠0, *F*_*y*_=0; likewise *E*_*y*_ to the condition *F*_*x*_=0, *F*_*y*_≠0. The displacements *r*_SP*x*_ and *r*_SP*y*_ of masses *m*_SP*x*_ and *m*_SP*y*_ from their equilibrium positions correspond to the induced dipole moments *d*_*x*_ and *d*_*y*_ in each particle, respectively. The equations of motion for the system are straightforward to derive but cumbersome, see Supplementary Note 1. Here we only discuss the main results.

**Figure 4:**
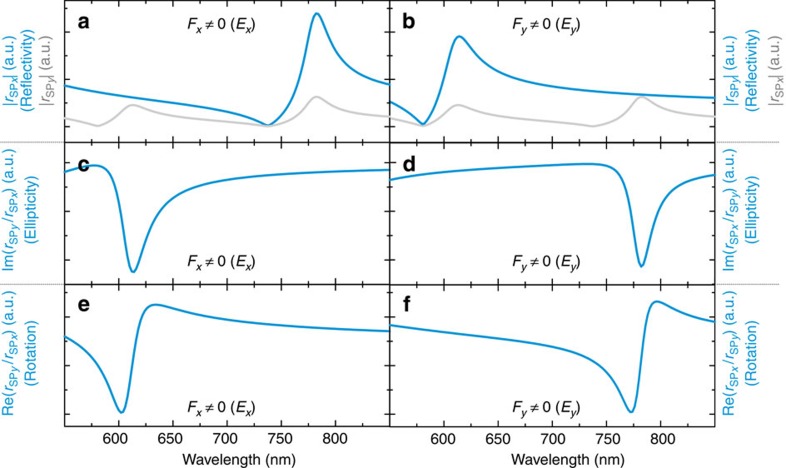
Results of the oscillator model. Blue lines: quantities analogous to the reflectivity (**a**,**b**), Kerr ellipticity (**c**,**d**) and Kerr rotation (**e**,**f**) as a function of *λ*. Here *λ* is defined as 2*πc*/*ω*, where *c* is the speed of light and *ω* is the angular frequency in the oscillator model. In **a**,**b**, the amplitude response of the non-driven oscillator is depicted as a grey line. The left column corresponds to *F*_*x*_≠0 (*E*_*x*_ polarization) and the right column to *F*_*y*_≠0 (*E*_*y*_ polarization).

We begin by inspecting the response for condition *F*_*x*_≠0, that is, *E*_*x*_ polarization (Fig. 4a,c,e). While the optical response |*r*_SP*x*_| exhibits Fano-like features at around 750 nm (Fig. 4a), the Kerr ellipticity and rotation (Im(*r*_SP*y*_/*r*_SP*x*_) and Re(*r*_SP*y*_/*r*_SP*x*_), respectively) exhibit features at 600 nm (Fig. 4c,e). This means that the optical response is governed by the period *p*_*y*_, while the magneto-optical response is sensitive to the period *p*_*x*_, which is in accordance with the experimental data (compare Fig. 4a,c,e with the blue lines in Fig. 2a,c,e respectively). Similarly, the condition *F*_*y*_≠0 (*E*_*y*_ polarization) results in an optical response at 600 nm dominated by *p*_*x*_, while the Kerr ellipticity and rotation responses at 750 nm are due to *p*_*y*_ (compare Fig. 4b,d,f with the blue lines in Fig. 2g,i,k, respectively).

We are able to explain this behaviour by analysing in detail the amplitude and phase response of the two oscillators *m*_SP*x*_ and *m*_SP*y*_. For simplicity, we focus on the case *F*_*x*_≠0 (the left column in Fig. 4). First, we note that while the amplitude response of the driven oscillator |*r*_SP*x*_| exhibits prominent features only at the wavelength corresponding to the SLR_*y*_ condition of the driven oscillator (the blue line in Fig. 4a), the non-driven oscillator |*r*_SP*y*_| exhibits nearly equally strong features at two wavelengths, one corresponding to resonance condition of SLR_*x*_ and the other to SLR_*y*_ (grey line in Fig. 4a). Thus, one would expect also the magneto-optically relevant ratio, *r*_SP*y*_/*r*_SP*x*_ to exhibit features at two wavelengths corresponding to SLR_*x*_ and to SLR_*y*_. The analytical expression for *r*_SP*y*_/*r*_SP*x*_, however, proves that this assumption is incorrect: for *F*_*x*_≠0, the ratio *r*_SP*y*_/*r*_SP*x*_ does not depend on any of the parameters related to the SLR_*y*_, that is, (*m*, *k*, *γ*)_SP*x*_, (*m*, *k*, γ)_DO*y*_ or *k*_RAD*y*_. Instead, we find that *r*_SP*y*_/*r*_SP*x*_=*A*/(*B*+*C*+*D*), where


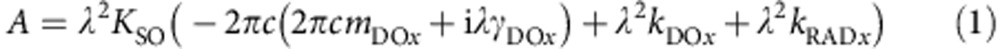



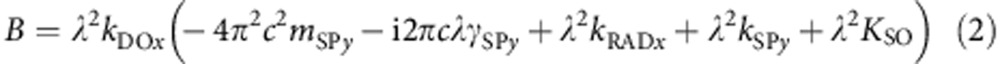



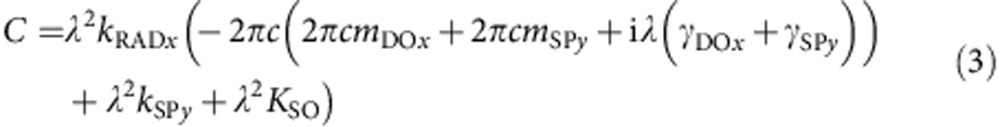



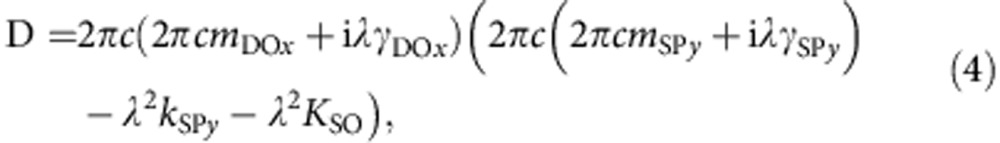


where *λ* is defined as 2*πc*/*ω*, here *c* is the speed of light and *ω* is the angular frequency in the oscillator model.

The complete absence of an SLR_*y*_-related magneto-optical signal at 750 nm is explained by the observation that the SLR_*y*_ enhances both *r*_SP*x*_ and *r*_SP*y*_ and that these changes exactly cancel each other in the ratio *r*_SP*y*_/*r*_SP*x*_ (Fig. 4c,e). A similar result is obtained for *F*_*y*_≠0; in this case, all parameters on the right of the spring *K*_so_ have no effect on the magneto-optical response, that is, on the ratio *r*_SP*x*_/*r*_SP*y*_, and correspondingly, no features are seen in the Kerr ellipticity and rotation at the SLR_*x*_ resonance wavelength of 600 nm (Fig. 4d,f).

To summarize, the physical origin of the purely optical response is threefold: (1) the individual particle polarizability along the direction of polarization (defined by (*m*, *k*, *γ*)_SP*x*_ for *F*_*x*_≠0); (2) the radiation predominantly along the direction perpendicular to the polarization (defined by *k*_RAD*y*_ for *F*_*x*_≠0); and (3) the radiation-induced hybridization of the single-particle resonances with the diffracted orders (defined by (*m*, *k*, *γ*)_DO*y*_ for *F*_*x*_≠0). In this simple model, the above parameters have no effect on the magneto-optical response. In contrast, the terms (*m*, *k*, *γ*)_SP*y*_, *k*_RAD*x*_ and (*m*, *k*, *γ*)_DO*x*_, affect the ratio *r*_SP*y*_/*r*_SP*x*_ and thus the magneto-optical response, but they have no effect on the purely optical response (that is, to |*r*_SP*x*_| when *F*_*x*_≠0).

## Discussion

In summary, we have shown that two-dimensional rectangular Ni nanoparticle arrays display SLRs leading to a spectrally narrow and strong magneto-optical response. While in the case of randomly arranged particles, the high ohmic losses of Ni result in very broad resonances, the arrays produce narrow spectral features (< 50 nm) due to hybridization of the LSPR with low-loss lattice modes. In a single nanoparticle, spin–orbit coupling induces optical dipoles in two orthogonal directions. In an array, we show that the intricate interplay between the two principal lattice directions results in a polar magneto-optical Kerr response that is governed by the lattice period parallel with the electric driving field, opposite to the purely optical response which is dominated by the period orthogonal to the driving field. The ability to accurately control longitudinal and orthogonal LSPRs by variation of the period and the spectral separation of SLRs provides highly tunable, narrow and intense magneto-optical resonances. At specific wavelengths, the Kerr ellipticity and rotation are enhanced four- and threefold, respectively, as compared with the case of randomly oriented particles.

Our results are different from the effects of periodic structures in magneto-plasmonics reported earlier. Either the systems reported were different (for example, a uniform film of ferromagnetic material overlaid with a one-dimensional gold grating^3^,^4^,^5^,^6^) or did not show the same results presented here. For instance, in the case of two-dimensional magnetic nanoparticle arrays^31^,^32^,^33^, the hexagonal lattice geometry did not allow for a direct assessment of the effects discussed here. The rectangular square lattice studied in ref. 36 was composed of gold/nickel composite particles, masking the effects arising from the pure plasmonic response of the magnetic material. Further, the array period was chosen such that the resonances we find were not present. In ref. 37, the effects of periodicity on magneto-optical activity in a magnetic nanowire array were studied—but only theoretically and for a system different from ours, namely, one-dimensional wires composed of dielectric material.

As the optical and magneto-optical responses of the system can be tuned independent of each other, one may consider further enhancing the magneto-optical activity without affecting the optical response. The analytical model provides the general method for doing so—parameters of the non-driven oscillator (SLR_*x*(*y*)_ for *E*_*x*(*y*)_ polarization) should be optimized in such a way that the ratio *r*_SP*y*_/*r*_SP*x*_ is maximized. This can be done (for *E*_*x*_ polarization of the incident field), for example, by bringing the resonance wavelengths of the LSPR_*y*_ and the diffracted order DO_*x*_ (see Fig. 3) close to each other. Another approach would be to further break the symmetry of the system—the resonances of plasmonic nanoparticle dimers are known to hybridize via optical nearfields, and, when the polarization of the driving field is parallel with the axis of the dimer, high-field enhancements and dipole moments are obtained at the resonance frequency. In our case, a single particle in the lattice could be replaced with a closely spaced dimer with axis oriented perpendicular to the polarization of the driving field, resulting in a higher magneto-optically induced dipole moment and thus enhanced magneto-optical activity. Furthermore, the magneto-optical response can be enhanced by reducing the losses. One might consider minimizing the radiative losses by hybridizing the lattice mode with a subradiant one, as is done for example, in ref. 26. Also, ohmic losses could be optimized on a given frequency regime by appropriate material choice (small imaginary part of the dielectric function at the desired wavelength range).

Our results are likely to initiate fruitful new research directions in magneto-plasmonics and magneto-optics. For instance, the nanoparticle geometry can be changed at will allowing for the design of dispersions, line widths and positions of the resonances. The SLR modes can be made highly coherent and may couple strongly with emitters, for instance; combining this with magnetic control opens up new future perspectives, for example, for information processing. The spectral selectivity, tunability as well as strength of the magneto-optical resonances found in our study also enable immediate and important applications, such as label-free phase-sensitive biosensing^38^. Our results are an example of how symmetry breaking at the material level (spin–orbit coupling) may interplay with the geometry and (a)symmetries of ordered nanostructures composed of the same material.

## Methods

### Sample fabrication

Cylindrical Ni nanoparticles in both random and periodic arrangements were fabricated by electron-beam lithography (Fig. 1a) on a glass substrate (Agar Scientific Microscope Cover glasses). Three different particle diameters were studied, namely 80, 120 and 160 nm while the thickness was maintained at 90 nm. All the measurements were carried in a symmetric refractive index (*n*=1.51) environment. The lattice constant for periodic arrays was varied between 400 and 500 nm, which places the Rayleigh anomalies in the 600–750 nm wavelength range. The dimensions for all arrays were (600 μm)^2^.

### Measurements

The optical responses of the samples were measured in the absence of external magnetic field. Zero-order reflection and transmission spectra of the samples were measured with an optical microscope (Nikon TE 2000) whose output was directed to a spectrometer (Princeton Instruments Acton SP2500). Angle-resolved transmission spectra for each array were measured by using the same setup and placing the back focal plane of the sample at the entrance slit of the spectrometer. The magneto-optical Kerr effect measurements were conducted with a Kerr spectrometer consisting of a broadband supercontinuum laser (NKT SuperK EXW-12 with acousto-optical filter), providing monochromatic light with a wavelength between 450 and 850 nm, polarizing optics, a photoelastic modulator (Hinds Instruments I/FS50), and a photodiode detector. The light was focused onto the sample at an angle of 0.5° with respect to the surface normal. The reflected light (diameter ∼1.5 mm) was passed through an aperture to spatially select light from the nanoparticle array only. Out-of-plane magnetic fields of up to ±400 mT were applied using an electromagnet (GMW Model 3470). The Kerr ellipticity and Kerr rotation were simultaneously recorded by lock-in amplification of the modulated signal at 50 and 100 kHz, respectively (see Supplementary Figs 8 and 9 and Supplementary Note 2). Magneto-optical spectra were obtained by measuring a hysteresis curve at each wavelength. From the curves, the saturated Kerr ellipticity and Kerr rotation could be accurately extracted by data averaging. During measurements, the samples were immersed in index matching oil to provide a symmetric refractive index environment. To contain the oil, a cover glass was used. Reflections from the front and back glass interfaces were eliminated by a background measurement of the bare glass system (that is, without Ni nanoparticles) and by placing a wedge prism on the backside of the glass substrate.

### Numerical methods

The magneto-optical responses of Ni arrays were modeled using DDA^28^,^29^,^30^. First, the polarizabilities *α* of single Ni scatterers were calculated using the measured permittivity *ɛ* of Ni^39^. The particles were assumed to be surrounded by homogeneous media with refractive index of 1.51. Owing to the comparably large sizes of individual scatterers, possible depolarization effects were taken into account by using the approach of ref. 12. The fabricated nanodisks were approximated as ellipsoidal particles of similar dimensions. The resulting polarizability tensor *α*_*ij*_ had the usual anti-symmetric structure of polar magneto-optical Kerr effect:


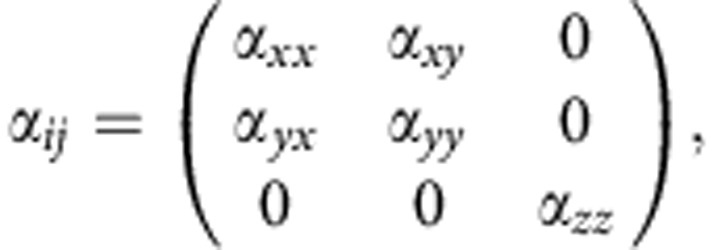


where for our case of cylindrically symmetric particles *α*_*xx*_=*α*_*yy*_ and *α*_*xy*_=−*α*_*yx*_ due to the external magnetizing field. The Cartesian coordinates correspond to the sample coordinates shown in Fig. 1.

A plane wave with two polarizations (*x* and *y*) at normal incidence was used for excitation. We found it computationally more efficient to simulate the collective responses of large arrays by simulating infinitely large arrays using periodic Green's functions^40^. The evaluation of periodic Green's functions was accelerated using the Ewald method^41^,^42^. We used the DDA formulation to solve the self-consistent internal field **E**_loc_, and wrote the algorithm using MATLAB. Since the measurements were performed in reflection geometry at normal incidence, the induced dipole moments were used to calculate scattered field in a backward direction using ref. 30


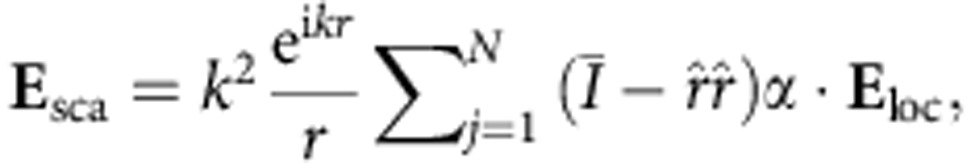


where *k* is the amplitude of the wave vector, 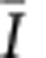 is the identity dyadic and 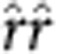 is the dyadic product of radial unit vector 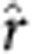. DDA calculations for Ni arrays were performed with a periodicity of *p*_*x*_=400 nm in *x* direction and periodicities of *p*_*y*_=400, 460, 480 and 500 nm in the *y* direction, as defined by the coordinate system shown in Fig. 1. Reflection spectra were calculated by taking the modulus squared of the scattered field, and the magneto-optical spectra were calculated from the ratios of the scattered fields, which in our case are equal to taking the ratios of induced polarizations **P**=*α***E**_loc_ for both input polarizations. Then, by taking the real (imaginary) part of the ratio


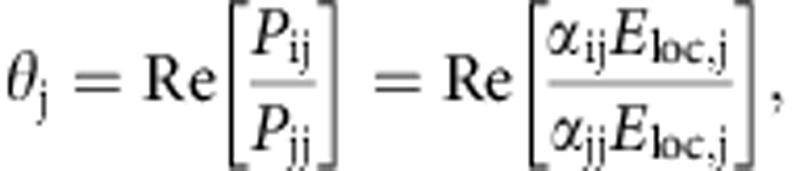



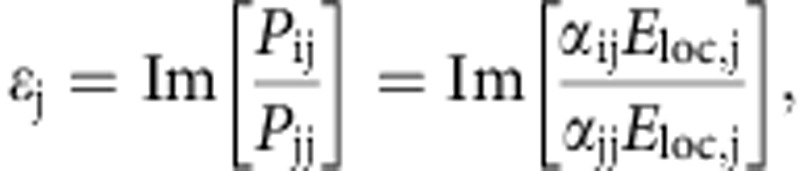


we obtain quantities proportional to the measured Kerr rotation (ellipticity).

The particles support also quadrupole modes but we have confirmed by FDTD simulations that those occur at much higher frequencies than the dipole resonances.

## Author contributions

P.T. and S.v.D. conceived the idea and supervised the project. M.K., T.K.H. and A.J. designed and performed the experiments and analysed the data. T.K.H., P.T. and A.J. provided the analytical model. M.J.H. provided the DDA model. A.J., M.K. and T.K.H. fabricated the samples. A.J. performed the FDTD simulations. All authors discussed the results. T.K.H., M.K. and P.T. wrote the manuscript together with all authors.

## Additional information

**How to cite this article:** Kataja, M. *et al*. Surface lattice resonances and magneto-optical response in magnetic nanoparticle arrays. *Nat. Commun.* 6:7072 doi: 10.1038/ncomms8072 (2015).

## Supplementary Material

Supplementary Information Supplementary Figures 1-9, Supplementary Table 1, Supplementary Notes 1-2 and Supplementary References

## References

[b1] Jain P. K., Xiao Y. H., Walsworth R. & Cohen A. E. Surface plasmon resonance enhanced magneto-optics (SuPREMO): Faraday rotation enhancement in gold-coated iron oxide nanocrystals. Nano Lett. 9, 1644–1650 (2009). 19351194 10.1021/nl900007k

[b2] Temnov V. V. . Active magneto-plasmonics in hybrid metal–ferromagnet structures. Nat. Photonics 4, 107–111 (2010).

[b3] Belotelov V.I. . Enhanced magneto-optical effects in magnetoplasmonic crystals. Nat. Nanotechnol. 6, 370–376 (2011). 21516090 10.1038/nnano.2011.54

[b4] Kreilkamp L. E. . Waveguide-plasmon polaritons enhance transverse magneto-optical Kerr effect. Phys. Rev. X 3, 041019 (2013).

[b5] Chin J. Y. . Nonreciprocal plasmonics enables giant enhancement of thin-film Faraday rotation. Nat. Commun. 4, 1599 (2013). 23511464 10.1038/ncomms2609

[b6] Belotelov V.I. . Plasmon-mediated magneto-optical transparency Nat. Commun. 4, 2128 (2013). 10.1038/ncomms3128 PMC3717503 23839481

[b7] Armelles G., Cebollada A., García-Martín A. & González M. U. Magnetoplasmonics: combining magnetic and plasmonic functionalities. Adv. Opt. Mater. 1, 10–35 (2013).

[b8] Banthí J. C. . High magneto-optical activity and low optical losses in metal-dielectric Au/Co/Au-SiO2 magnetoplasmonic nanodisks. Adv. Mater. 24, OP36–OP41 (2012). 22213149 10.1002/adma.201103634

[b9] González-Díaz J. B. . Enhanced magneto-optics and size effects in ferromagnetic nanowire arrays. Adv. Mater. 19, 2643–2647 (2007).

[b10] Chen J. . Plasmonic nickel nanoantennas. Small 7, 2341 (2011). 21678553 10.1002/smll.201100640

[b11] Bonanni V. . Designer magnetoplasmonics with nickel nanoferromagnets. Nano Lett. 11, 5333–5338 (2011). 22029387 10.1021/nl2028443 PMC3238448

[b12] Maccaferri N. . Polarizability and magnetoplasmonic properties of magnetic general nanoellipsoids. Opt. Express 21, 9875–9889 (2013). 23609693 10.1364/OE.21.009875

[b13] Maccaferri N. . Tuning the magneto-optical response of nanosize ferromagnetic Ni disks using the phase of localized plasmons. Phys. Rev. Lett. 111, 167401 (2013). 24182300 10.1103/PhysRevLett.111.167401

[b14] Markel V. Coupled-dipole approach to scattering of light from a one-dimensional periodic dipole structure *J*. Modern Opt. 40, 2281–2291 (1993).

[b15] Zou S., Janel N. & Schatz G. C. Silver nanoparticle array structures that produce remarkably narrow plasmon lineshapes. J. Chem. Phys. 120, 10871–10875 (2004). 15268116 10.1063/1.1760740

[b16] García de Abajo F. J. Colloquium: light scattering by particle and hole arrays. Rev. Mod. Phys. 79, 1267 (2007).

[b17] Kravets V. G., Schedin F. & Grigorenko A. N. Extremely narrow plasmon resonances based on diffraction coupling of localized plasmons in arrays of metallic nanoparticles. Phys. Rev. Lett. 101, 087403 (2008). 18764660 10.1103/PhysRevLett.101.087403

[b18] Auguié B. & Barnes W. L. Collective resonances in gold nanoparticle arrays. Phys. Rev. Lett. 101, 143902 (2008). 18851529 10.1103/PhysRevLett.101.143902

[b19] Chu Y., Schonbrun E., Yang T. & Crozier K. B. Experimental observation of narrow surface plasmon resonances in gold nanoparticle arrays. Appl. Phys. Lett. 93, 181108 (2008).

[b20] Rodriguez S. R. K. . Coupling bright and dark plasmonic lattice resonances. Phys. Rev. X 1, 021019 (2011).

[b21] Väkeväinen A. I. . Plasmonic surface lattice resonances at the strong coupling regime. Nano Lett. 14, 1721–1727 (2014). 24279840 10.1021/nl4035219

[b22] Shi L. . Spatial coherence properties of organic molecules coupled to plasmonic surface lattice resonances in the weak and strong coupling regimes. Phys. Rev. Lett. 112, 153002 (2014). 24785036 10.1103/PhysRevLett.112.153002

[b23] Törmä P. & Barnes W. L. Strong coupling between surface plasmon polaritons and emitters: a review. Rep. Prog. Phys. 78, 013901 (2015). 25536670 10.1088/0034-4885/78/1/013901

[b24] Vitrey A., Aigouy L., Prieto P., García-Martín J. M. & González M. U. Parallel collective resonances in arrays of gold nanorods. Nano Lett. 14, 2079–2085 (2014). 24645987 10.1021/nl500238h

[b25] Nikitin A. G. Diffraction-induced subradiant transverse-magnetic lattice plasmon modes in metal nanoparticle arrays. Appl. Phys. Lett. 104, 061107 (2014).

[b26] Odom T. & Zhou W. Tunable subradiant lattice plasmons by out-of-plane dipolar interactions. Nat. Nanotechnol. 6, 423–427 (2011). 21572429 10.1038/nnano.2011.72

[b27] Zhou W. . Lasing action in strongly coupled plasmonic nanocavity arrays. Nat. Nanotechnol. 8, 506–511 (2013). 23770807 10.1038/nnano.2013.99

[b28] Yurkin M. A. & Hoekstra A. G. The discrete dipole approximation: an overview and recent developments. J. Quant. Spectrosc. Ra. 106, 558–589 (2007).

[b29] Purcell E. & Pennypacker C. Scattering and absorption of light by nonspherical dielectric grains. Astrophys J. 186, 705–714 (1973).

[b30] Draine B. T. & Flatau P. J. Discrete-dipole approximation for scattering calculations. J. Opt. Soc. Am. A 11, 1491–1499 (1994).

[b31] Chetvertukhin A. V., Grunin A. A., Dolgova T. V., Inoue M. & Fedyanin A. A. Transversal magneto-optical Kerr effect in two-dimensional nickel magnetoplasmonic crystals. J. Appl. Phys. 113, 17A942 (2013).

[b32] Chetvertukhin A.V. . Magneto-optical Kerr effect enhancement at the Wood's anomaly in magnetoplasmonic crystals. J. Magn. Magn. Mater. 324, 3516–3518 (2012).

[b33] Papaioannou E. T., Meyer T. & Hillebrands B. Magneto-optical enhancement in Co/Au patterned nanostructures. J. Surf. Interface. Mater. 2, 40–45 (2014).

[b34] Armelles G. . Mimicking electromagnetically induced transparency in the magneto-optical activity of the magnetoplamonic nanoresonators. Opt. Express 21, 27356 (2014). 24216958 10.1364/oe.21.027356

[b35] de Sousa N. . Interaction effects on the magneto-optical response of magnetoplasmonic dimers. Phys. Rev. B 89, 205419 (2014).

[b36] Du G. X., Saito S. & Takahashi M. Spectroscopic characterization of magnetoplasmonic nanodisk array: size, shape and lattice constant. Preprint at http://arxiv.org/abs/1306.1451 (2013).

[b37] Marinchio H., Carminati R., García-Martín A. & Sáenz J. J. Magneto-optical Kerr effect in resonant subwavelength nanowire gratings. New J. Phys. 16, 015007 (2014).

[b38] Maccaferri N. . Ultrasensitive and label-free molecular-level detection enabled by light phase control in magnetoplasmonic nanoantennas. Nat. Commun. 6, 6150 (2015). 25639190 10.1038/ncomms7150 PMC4340560

[b39] Krinchik G. S. & Artemjev V. A. Magnetooptic properties of nickel, iron, and cobalt. J. Appl. Phys. 39, 1276–1278 (1968).

[b40] Valerio G., Baccarelli P., Burghignoli P. & Galli A. Comparative analysis of acceleration techniques for 2-D and 3-D Green's functions in periodic structures along one and two directions. IEEE Trans. Antennas. Propag. 55, 1630–1643 (2007).

[b41] Ewald P. P. Die Berechnung optischer und elektrostatischer Gitterpotentiale. Ann. Phys. vol. 64, pp 253–287 (1921), Translated by Cornell A. Atomics International Library (1964).

[b42] Capolino F., Wilton D. R. & Johnson W. A. Efficient computation of the 3D Green's function for the Helmholtz operator for a linear array of point sources using the Ewald method. J. Comput. Phys. 223, 250–261 (2007).

