# Supplementary material for: Surface lattice resonances and magneto-optical response in magnetic nanoparticle arrays
Source: Nat Commun. 2015 May 7;6:7072. doi: 10.1038/ncomms8072 (PMC4432637; doi:10.1038/ncomms8072)
Supplement: Supplementary Information — Supplementary Figures 1-9, Supplementary Table 1, Supplementary Notes 1-2 and Supplementary References [file ncomms8072-s1.pdf]

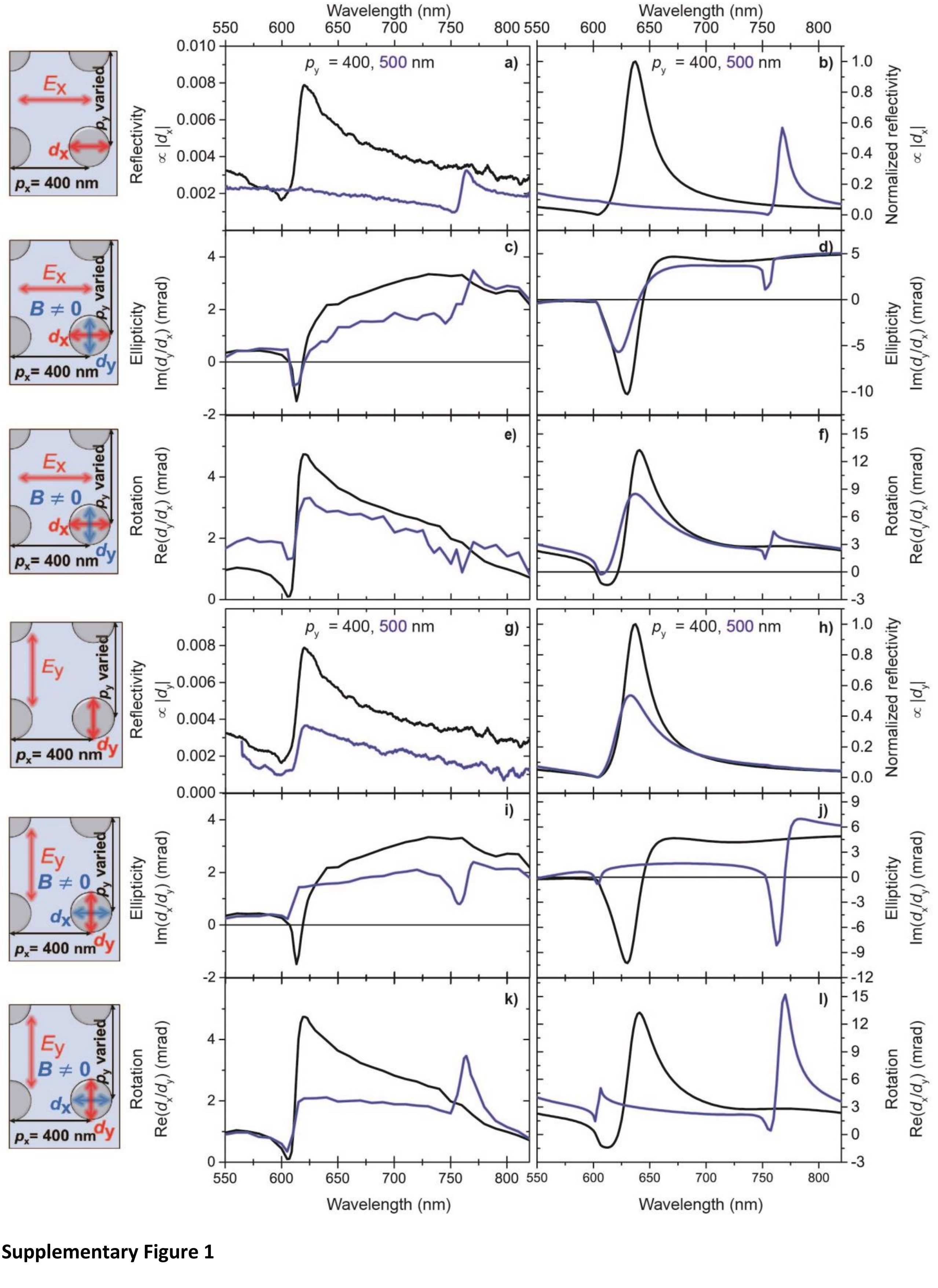

Supplementary Figure 1

Optical and magneto-optical responses for 80 nm diameter particles

The schematics on the left illustrate the direction of incident polarization and the induced dipole moments that are probed in the measurements. The graphs on the left (right) show experimental (DDA) results for the 80 nm particles. The black line corresponds to $p_x = p_y = 400$ nm sample and the blue line to $p_x = 400$ nm, $p_y = 500$ nm sample (a and b) Normal incidence optical reflectivity for incident light polarization E_x. The magneto-optical Kerr ellipticity (c and d) and rotation (e and f) with polarization E_x. Normal incidence optical reflectivity (g and h), the magneto-optical Kerr ellipticity (i and j) and rotation (k and l) for polarization E_y. Note, that similar to the data presented for the 120 nm diameter particles in the main manuscript, the DDA simulations were carried out with a small (0.04 degree) sample rotation.

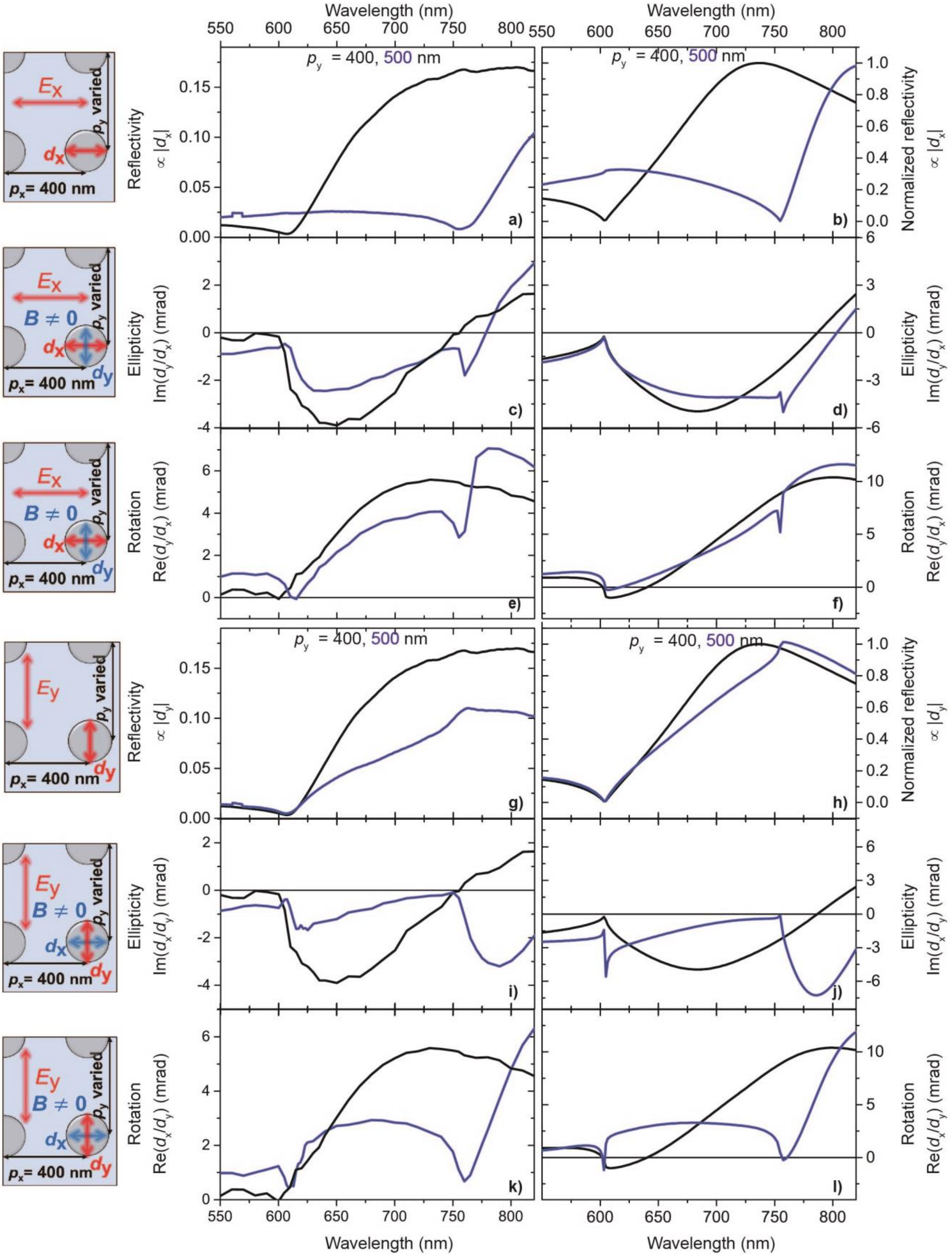

Supplementary Figure 2

Optical and magneto-optical responses for 160 nm diameter particles

The schematics on the left illustrate the direction of incident polarization and the induced dipole moments that are probed in the measurements. The experimental data (left column) and results from DDA simulations (right column). The black line corresponds to $p_x = p_y = 400$ nm sample and the blue line to $p_x = 400$ nm, $p_y = 500$ nm sample. (a and b) Normal incidence optical reflectivity for incident light polarization E_x. The magneto-optical Kerr ellipticity (c and d) and rotation (e and f) with polarization E_x. Normal incidence optical reflectivity (g and h), the magneto-optical Kerr ellipticity (i and j) and rotation (k and l) for polarization E_y. For the 160 nm diameter particles, the DDA simulations were carried out with a (0.02 degree) rotation of incident light polarization.

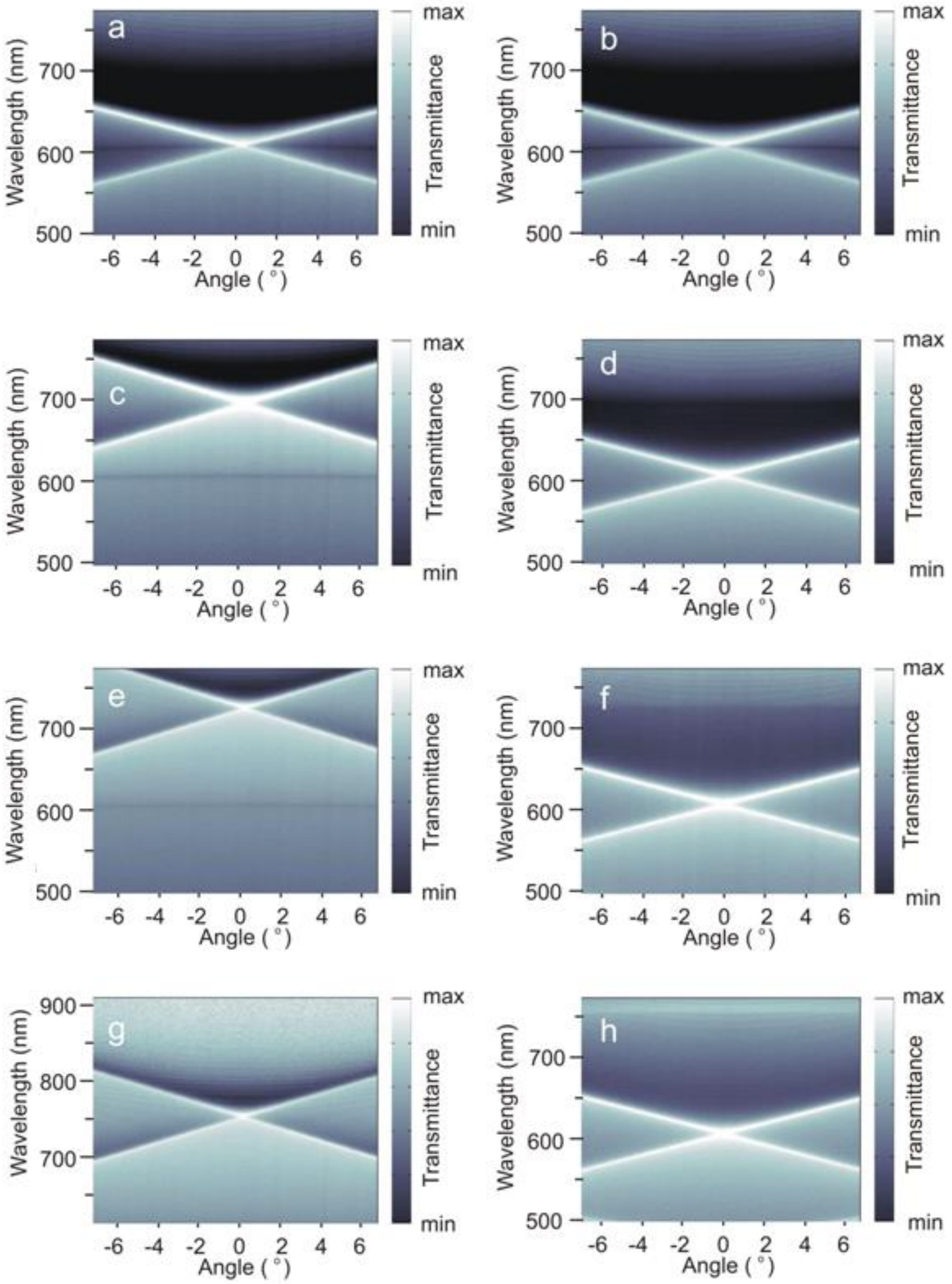

Supplementary Figure 3

Angle and wavelength dependent transmission curves

To study the optical modes of the samples, angle and wavelength dependent transmissions of the samples were measured with two polarizations E_x (left column) and E_y (right column). Periodicity p_x = 400 nm and p_y = 400 nm (a, b), 460 nm (c, d), 480 nm (e, f), and 500 nm (g, h). Note the different wavelength scale in (g).The linear dispersions of the diffracted <+1, 0> and <-1, 0> orders of the lattice are clearly visible in each plot. Note that with E_x polarization, the crossing point gradually redshifts from 600 nm to 750 nm with increasing periodicity p_y (figures a, c, e and g) but with E_y polarization the crossing point remains at 600 nm (figures b, d, f and h).

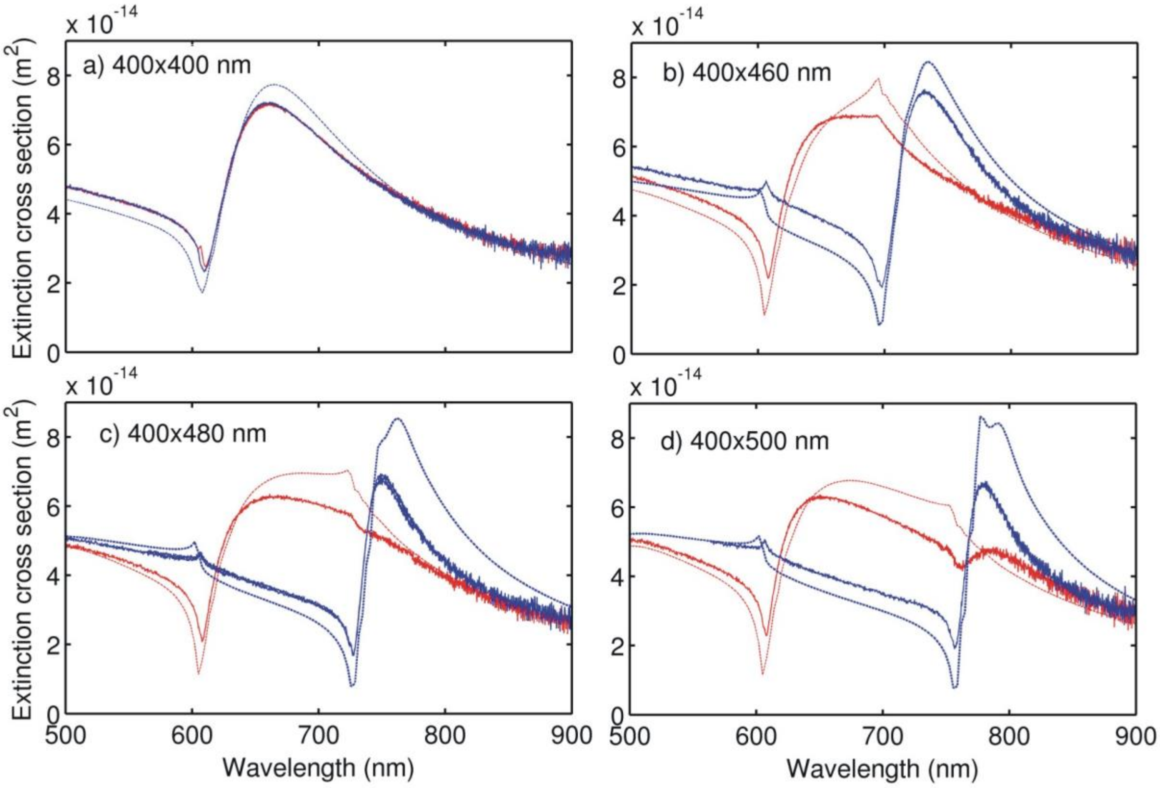

Supplementary Figure 4

Normal incidence extinction curves (defined as 1-T) for samples with different periodicities

a) p_x= 400 nm, p_y = 400 nm, b) 460 nm, c) 480 nm, and d) 500 nm. The blue (red) lines correspond to the E_x (E_y) polarization in all the figures. The solid lines correspond to measured extinction, while the dashed lines correspond to extinction obtained from the FDTD simulations. The FDTD simulations were carried out under similar conditions as the experiments: we used cylindrical particles with diameter 120 nm and height 90 nm with optical parameters of nickel (Ni) obtained from [1]. The refractive index of n = 1.5 was used. Importantly, in the simulation only one Ni particle was used with periodic boundary conditions in x and y direction that were matched with the periodicities p_x and p_y. The good agreement between experiments and simulations further supports our conclusion that the optical response is indeed dominated by the periodicity perpendicular to the incident polarization.

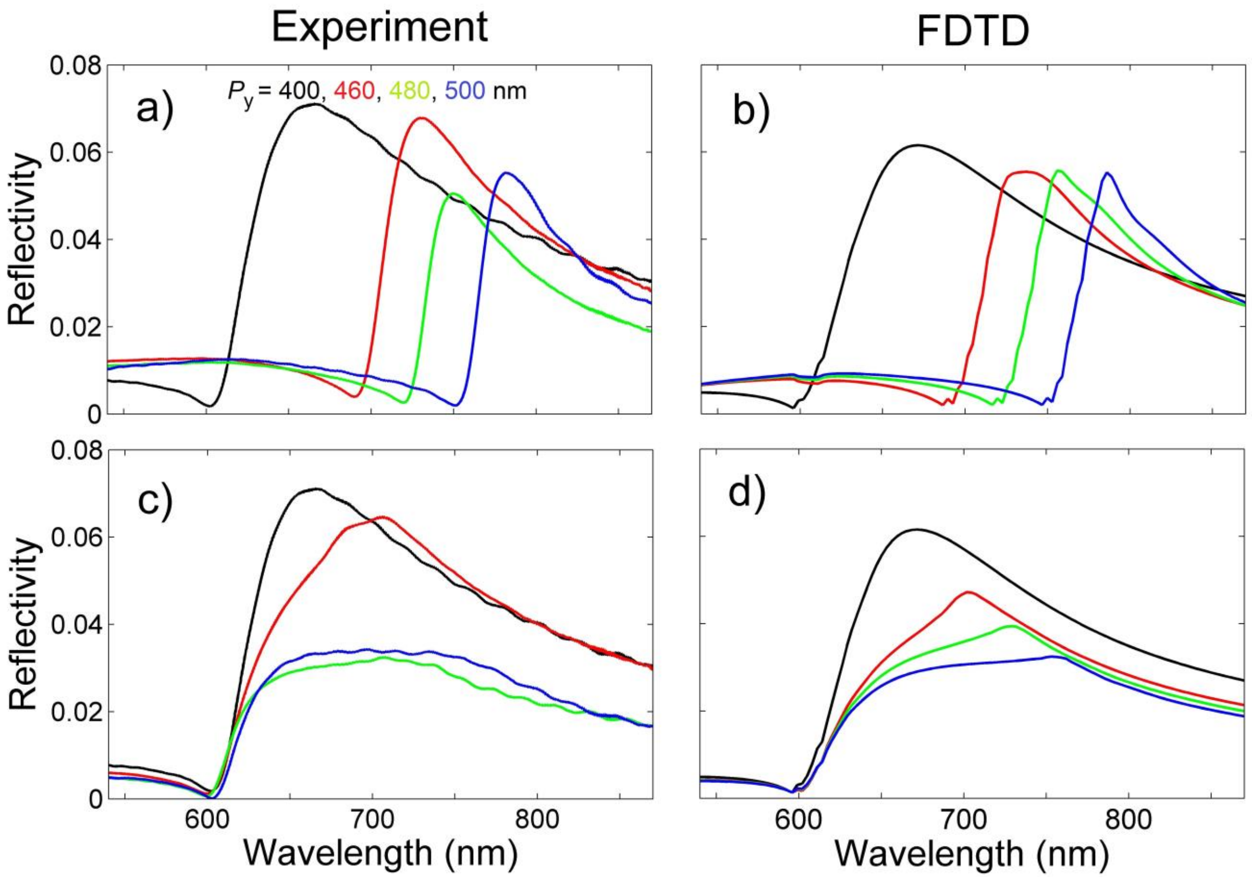

Supplementary Figure 5

The measured zero-order reflectivities and the corresponding simulations

a) The measured zero-order reflectivity with polarization E_x for samples having p_x = 400 nm and p_y = 400 nm, 460 nm, 480 nm, and 500 nm (black, red green and blue lines, respectively), and b) the corresponding FDTD simulation. c) The measured zero order reflectivity with polarization E_y and d) the corresponding FDTD simulation. The color coding and the *y*-axis scale is the same in all the figures.

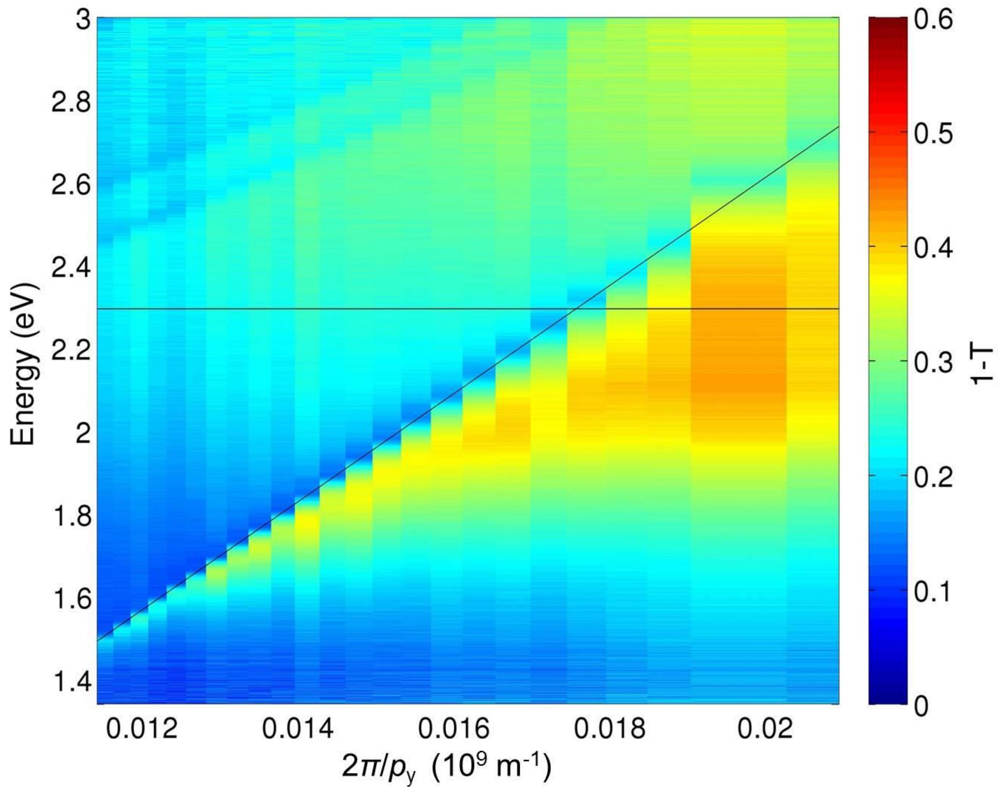

Supplementary Figure 6

Determining the coupling strength between the localized surface plasmon resonance (LSPR) and the diffracted orders of the Ni nanoparticle lattice

The normal incidence extinction simulated using the FDTD method is plotted as functions of energy and the inverse lattice constant $2\pi/p_y$. The polarization was E_x and sample periodicity p_x was maintained at 400 nm while p_y was varied from 300 nm to 550 nm. The black horizontal line corresponds to Ni particle (diameter 120 nm, height 90 nm) LSPR, and the tilted line to the crossing of the <+1, 0> and <-1, 0> diffracted orders of the lattice. From the above simulation, one can estimate the coupling strength between the localized surface plasmon resonance (LSPR) and the diffracted orders of the periodic Ni arrays under normal incidence illumination. The horizontal black line corresponds to uncoupled LSPR of the Ni particle, and the tilted black line to the crossing point of the <+1, 0> and <-1, 0> diffracted orders of the lattice at a zero incidence angle. As can be seen, the maxima of the extinction clearly deviate from the uncoupled LSPR. The splitting is on the order of 400 meV.

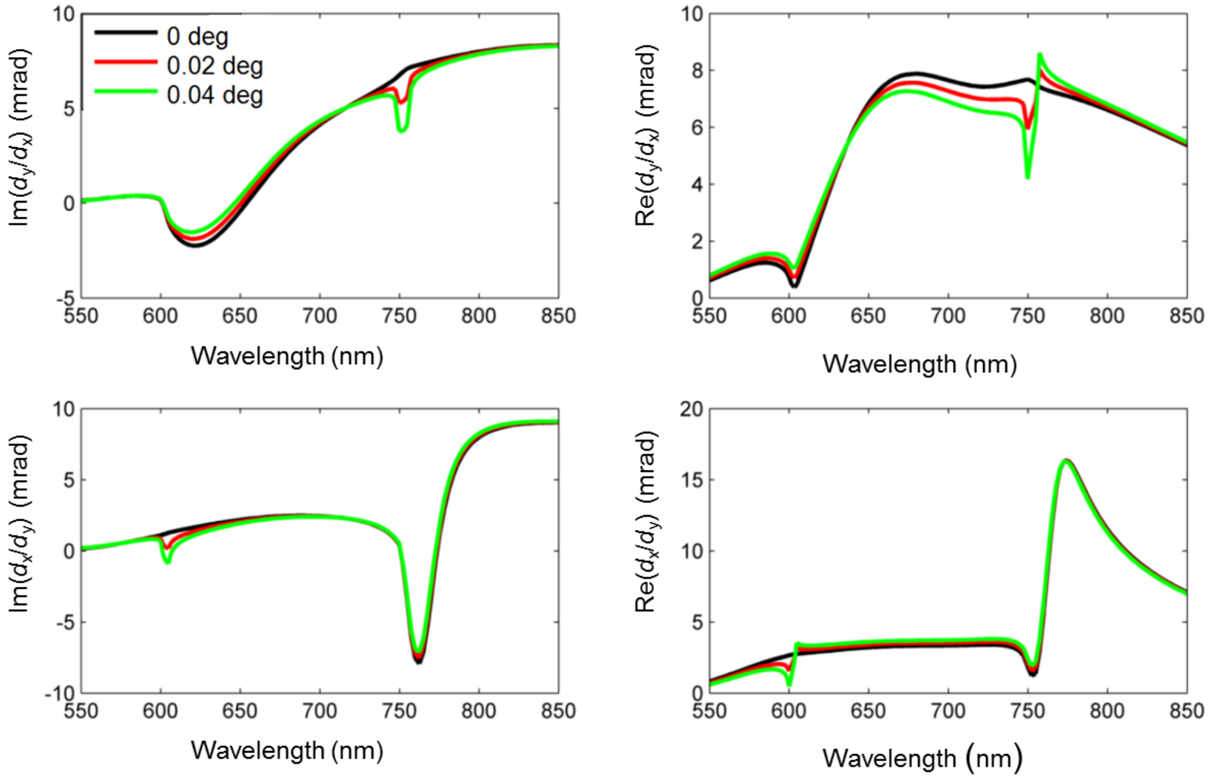

Supplementary Figure 7

Effect of small misalignment of the incident polarization for the simulated magneto-optical responses

Calculated Kerr a) ellipticities and b) rotations for ideally and almost *x*-polarized incident light. Kerr c) ellipticities and d) rotations for ideally and almost *y*-polarized incident light. Black lines correspond to the cases with ideal polarization. The red (green) line corresponds to a polarization rotated by 0.02 deg (0.04 deg) from the ideal direction. To fully understand the finer details of the measured magneto-optical responses, we also studied how small deviations from an ideal input polarization of the driving field can affect the measured responses. In the ideal case, the input polarizations for the driving field are along the array periodicities (in *x*- and *y*-directions). But in experimental conditions, the incident polarization is never purely linear and oriented along the periodicities. Therefore, we measured the extinction ratio between the unwanted and wanted polarization components, which was found to be in the order of 15 000:1 corresponding to a ~0.04 degree offset from the ideal direction.

We then simulated the effect of this offset to the magneto-optical responses by performing DDA simulations while rotating the incident polarization a bit. Example results for sample with p_x = 400 nm p_y = 500 nm for the ideal case and for angle offsets of 0.02 deg and 0.04 deg are shown in Supplementary Figure 7. Interestingly for the ideal case, the results are almost entirely governed by the SLR conditions related to the period *parallel* to the driving field. For the cases with small deviations in the input polarizations, the cross-coupling effects seen in experimental data (see Figure 2 of the manuscript) appear and increase as a function of the deviation angle giving better agreement between the simulations and the measurements. Therefore, we conclude that the finer details of the measured magneto-optical responses are due to a small input polarization impurity and a possible slight misalignment in the polarization.

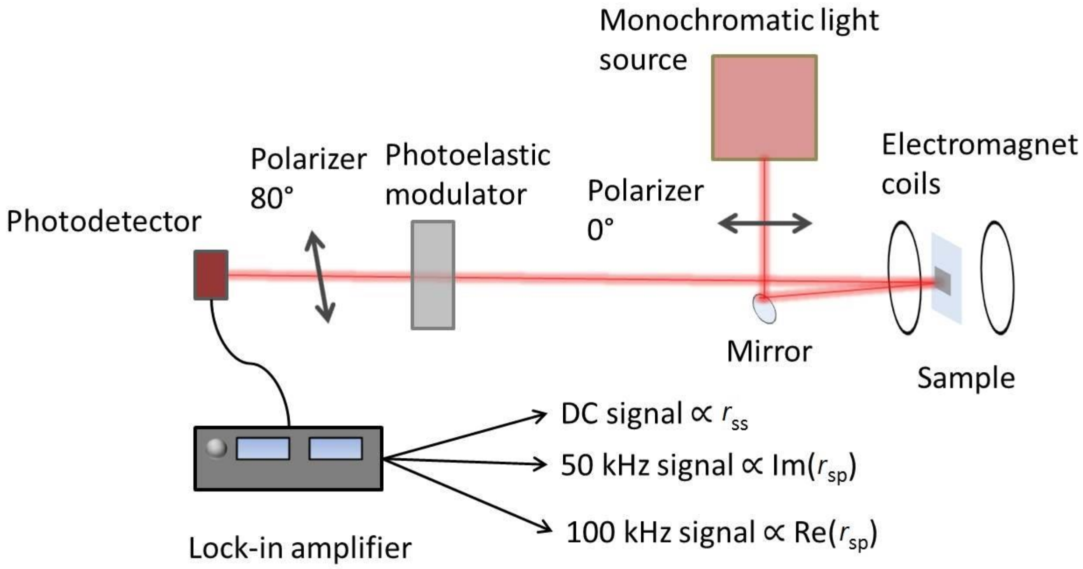

Supplementary Figure 8

Magneto-optical measurement setup

The Magneto-optical Kerr spectrometer consist of a broadband supercontinuum laser (NKT SuperK EXW-12 with acousto-optical filter), polarizing (Glann-Thompson prism, CVI Melles Griot) and focusing optics, a photoelastic modulator (Hinds Instruments I/FS50), and a photodetector. The wavelength of the laser is tunable between 450 nm and 850 nm. Measurements are performed in polar magneto-optical Kerr geometry. The angle of incident light is 0.5° with respect to the surface normal and an electromagnet (GMW 3470) is used to reorient the magnetization of the Ni nanoparticles between the two out-of-plane directions in a maximum field of ± 400 mT. The Kerr ellipticity and Kerr rotation are simultaneously recorded by lock-in amplification of the modulated signal at 50 kHz and 100 kHz (see Supplementary Note 2).

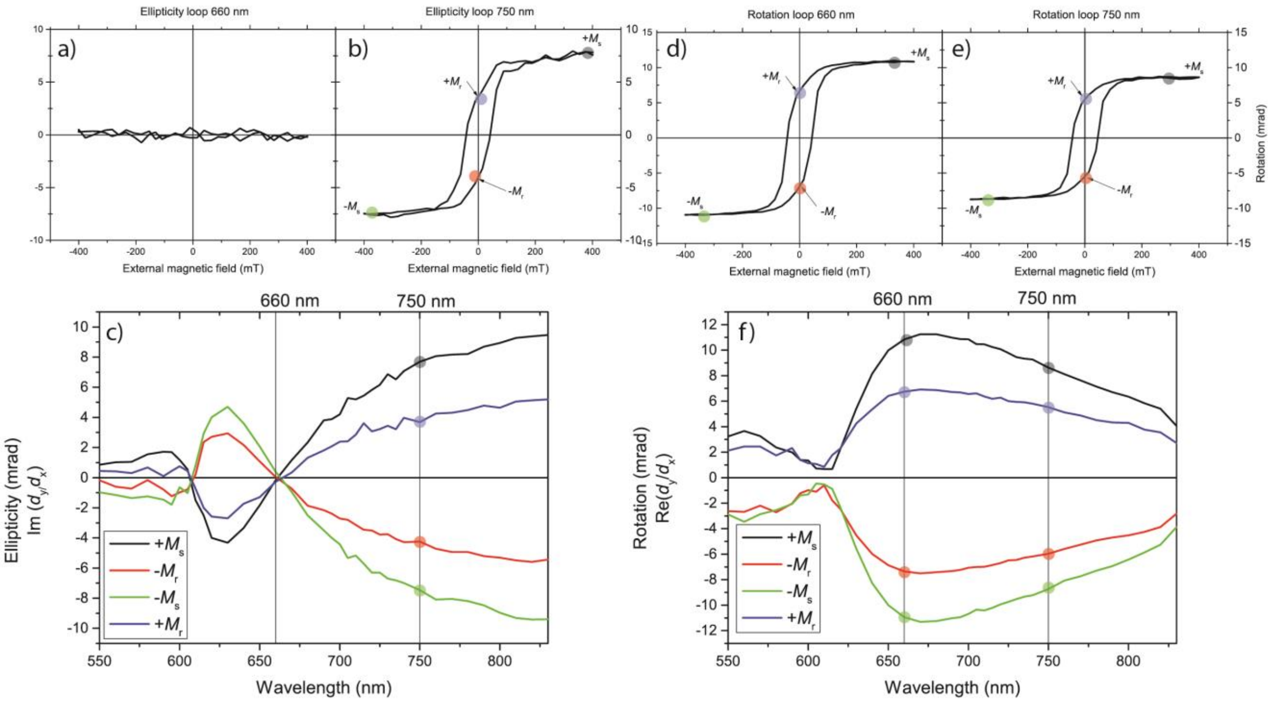

Supplementary Figure 9

Kerr ellipticity and Kerr rotation spectra

In the magneto-optical Kerr effect measurements, full polar hysteresis curves of the Kerr ellipticity and Kerr rotation are recorded for each wavelength. Examples of Kerr ellipticity measurements at 660 nm and 750 nm are shown in (a) and (b). Corresponding curves for the Kerr rotation are shown in (d) and (e). From a full set of hysteresis curves, the magneto-optical Kerr spectra are extracted. As an example, panels (c) and (f) display Kerr ellipticity and Kerr rotation spectra for different magnetization states in the Ni nanoparticles (remanence and saturation). In the main manuscript, only data for fully out-of-plane magnetization are discussed.

	(kg)
m_{SPx}	1
m_{SPy}	1
m_{DOx}	1
m_{DOy}	1

	(Hz)
γ_{SPx}	1.0×10^{15}
γ_{SPy}	1.0×10^{15}
γ_{DOx}	2.0×10^{13}
γ_{DOy}	1.0×10^{13}

	(kg s^{-2})
k_{SPx}	1.4×10^{31}
k_{SPy}	1.4×10^{31}
k_{DOx}	7.7×10^{30}
k_{DOy}	3.7×10^{30}

	(kg s^{-2})
k_{RADx}	2.8×10^{30}
k_{RADy}	2.8×10^{30}
K_{SO}	1.4×10^{27}

Supplementary Table 1

The parameters used in the coupled oscillator model

The model parameters have been obtained from the experimental data. In particular, resonance frequencies (as defined by $\sqrt{k/m}$) and linewidths (γ) of single particle resonances and diffractive orders have been chosen to match the experimentally obtained frequencies and linewidths. The single particle properties are obtained from measurements of the samples with random particle positions. The parameters related to the diffractive orders are obtained from optical transmission measurements. In our case of cylindrical particles, the two single particle resonances in *x*- and *y*-direction are equal, while the resonance frequencies of the diffractive modes in *x*- and *y*-direction can be different. Further, the linewidths of the diffractive orders have been chosen to be significantly smaller than the single particle resonances to account for the fact that these modes suffer much less from ohmic losses. The coupling strength of the diffractive orders to single particle resonances (i.e., the spring constants k_{RADy} and k_{RADx}) have been estimated from the extinction data obtained from the FDTD simulations under normal incidence with equal particle size and geometry with the experiments (see also Supplementary Figure 6). Finally, the coupling strength of the optically driven dipole to the magneto-optical one (value for K_{SO}) has been determined from the existing experimental off-diagonal polarizability elements for Ni [2].

Supplementary Note 1

The mathematical description of the coupled oscillator model

The equations of motion for the coupled oscillator model (see also Manuscript Figure 3) are

$$m_{\mathrm{DOy}}\ddot{r}_{\mathrm{DOy}} = -k_{\mathrm{DOy}}r_{\mathrm{DOy}} - \gamma_{\mathrm{DOy}}\dot{r}_{\mathrm{DOy}} - k_{\mathrm{RADy}}(r_{\mathrm{DOy}} - r_{\mathrm{SPx}})$$

$$m_{\mathrm{SPx}}\ddot{r}_{\mathrm{SPx}} = -k_{\mathrm{SPx}}r_{\mathrm{SPx}} - \gamma_{\mathrm{SPx}}\dot{r}_{\mathrm{SPx}} - K_{\mathrm{SO}}(r_{\mathrm{SPx}} - r_{\mathrm{SPy}}) - k_{\mathrm{RADy}}(r_{\mathrm{SPx}} - r_{\mathrm{DOy}}) + F_{\mathrm{x}}$$

$$m_{\mathrm{SPy}}\ddot{r}_{\mathrm{SPy}} = -k_{\mathrm{SPy}}r_{\mathrm{SPy}} - \gamma_{\mathrm{SPy}}\dot{r}_{\mathrm{SPy}} + K_{\mathrm{SO}}(r_{\mathrm{SPx}} - r_{\mathrm{SPy}}) - k_{\mathrm{RADx}}(r_{\mathrm{SPy}} - r_{\mathrm{DOx}}) + F_{\mathrm{y}}$$

$$m_{\mathrm{DOx}}\ddot{r}_{\mathrm{DOx}} = -k_{\mathrm{DOx}}r_{\mathrm{DOx}} - \gamma_{\mathrm{DOx}}\dot{r}_{\mathrm{DOx}} + k_{\mathrm{RADx}}(r_{\mathrm{SPy}} - r_{\mathrm{DOx}}).$$

The above equations can be solved with respect to r_{SPx} and r_{SPy} for both driving conditions ($F_{\mathrm{x}} \neq 0$ or $F_{\mathrm{y}} \neq 0$). The displacement r from the equilibrium position and the driving force F are assumed to be of the form $r = r_0\, e^{\mathrm{i}\omega t}$ and $F = F_0\, e^{\mathrm{i}\omega t}$, respectively. Here ω is the angular frequency. While both r_{SPx} and r_{SPy} have extremely complex expressions, it turns out that the ratio is significantly simpler, despite the inherent asymmetry in the system ($p_{\mathrm{x}} \neq p_{\mathrm{y}}$, thus the m_{DO}, k_{DO} and γ_{DO} will be different for each direction as well). In the case of $F_{\mathrm{x}} \neq 0$, the ratio between the displacements of the nondriven oscillator and the driven one can be written in the form $r_{\mathrm{SPy}} / r_{\mathrm{SPx}} = A/(B+C+D)$, where

$$A = \lambda^2 K_{\mathrm{SO}}(-2\pi c(2\pi c m_{\mathrm{DOx}} + \mathrm{i}\lambda\gamma_{\mathrm{DOx}}) + \lambda^2 k_{\mathrm{DOx}} + \lambda^2 k_{\mathrm{RADx}})$$

$$B = \lambda^2 k_{\mathrm{DOx}}(-4\pi^2 c^2 m_{\mathrm{SPy}} - \mathrm{i}2\pi c\lambda\gamma_{\mathrm{SPy}} + \lambda^2 k_{\mathrm{RADx}} + \lambda^2 k_{\mathrm{SPy}} + \lambda^2 K_{\mathrm{SO}})$$

$$C = \lambda^2 k_{\mathrm{RADx}}\left(-2\pi c\left(2\pi c m_{\mathrm{DOx}} + 2\pi c m_{\mathrm{SPy}} + \mathrm{i}\lambda(\gamma_{\mathrm{DOx}} + \gamma_{\mathrm{SPy}})\right) + \lambda^2 k_{\mathrm{SPy}} + \lambda^2 K_{\mathrm{SO}}\right)$$

$$\mathrm{D} = 2\pi c(2\pi c m_{\mathrm{DOx}} + \mathrm{i}\lambda\gamma_{\mathrm{DOx}})(2\pi c(2\pi c m_{\mathrm{SPy}} + \mathrm{i}\lambda\gamma_{\mathrm{SPy}}) - \lambda^2 k_{\mathrm{SPy}} - \lambda^2 K_{\mathrm{SO}}) .$$

Here λ is defined as $2\pi c / \omega$, where c is the speed of light. The expression for $F_y \neq 0$ is obtained upon switching the indices x and y. Interestingly, for $F_x \neq 0$ the ratio r_{SPy} / r_{SPx} defining the magneto-optical response, has *no dependence on any of the parameters* related to the SLR_y, i.e., $(m, k, \gamma)_{SPx}$, $(m, k, \gamma)_{DOy}$ or k_{RADy}. Note that in our model, all these parameters are on the left side of the central spring $K_{SO,}$ and in the absence of magnetic field ($K_{SO} = 0$), they are solely responsible for the purely optical response (i.e., the reflectivity and the value of $|r_{SPx}|$ in our model). In contrast, the terms $(m, k, \gamma)_{SPy}$, $(m, k, \gamma)_{DOx}$ and k_{RADx} (parameters are on the right side of the central spring K_{SO} in Manuscript Figure 3) do affect the ratio r_{SPy} / r_{SPx} and thus the magneto-optical response, but they have no effect on purely optical response (i.e., to $|r_{SPx}|$ when $F_x \neq 0$ and K_{so}=0).

By neglecting the radiation and periodicity induced effects, that is, by setting $k_{RADx(y)} = k_{DOx(y)} = 0$, one recovers a result for individual particle (or randomly distributed particles). In the case $F_x \neq 0$, the ratio becomes

$$\frac{r_{\text{SPy}}}{r_{\text{SPx}}} = \frac{\lambda^2 K_{\text{SO}}}{-2\pi c\left(2\pi c m_{\text{SPy}} + i\lambda\gamma_{\text{SPy}}\right) + \lambda^2 k_{\text{SPy}} + \lambda^2 K_{\text{SO}}}.$$

The ratio is again independent of the parameters of the driven oscillator m_{SPx} and is in agreement with the earlier studies made for individual Ni particles [3,4]. In this simple case, the physical interpretation is straightforward: The force felt by the non-driven oscillator m_{SPy} is linearly dependent on the displacement of the driven one, r_{SPx}, which in turn depends on the parameters on the left side of the central spring K_{SO}. Thus r_{Spy} depends also on these parameters, but in the ratio r_{Spy} / r_{Spx} these parameters on the left of the central spring K_{SO} cancel out.

Finally, we note that while the analytical model neglects a number of experimentally relevant parameters, such as the wavelength dependence of both the Ni optical parameters and the spin orbit coupling and the actual radiation pattern of the particles, it is nevertheless able to reproduce the most prominent features of the experimental data, namely 1) the dip in the ellipticity curve and 2) strongly asymmetric line shape in the rotation, both appearing at the resonance wavelength of the non-driven oscillator.

Supplementary Note 2

Jones matrix representation of the magneto-optical measurement setup

The components of the measurement setup (Supplementary Figure 8), including the magnetic nanoparticle sample, can be described by Jones matrices. The incident p-polarized light is represented by a column vector

$$\mathbf{E}_{\mathrm{inc}} = E_0 \begin{bmatrix} 1 \\ 0 \end{bmatrix},$$

where E_0 is the amplitude of the incoming electric field. The light reflected from the magnetic sample is represented by the matrix

$$S = \begin{bmatrix} r_{\mathrm{pp}} & r_{\mathrm{ps}} \\ r_{\mathrm{sp}} & r_{\mathrm{ss}} \end{bmatrix},$$

where the diagonal components represent the Fresnel reflection coefficients, i.e., the reflected light having the same polarization as the incident light. The off-diagonal components represent the magneto-optical effects, i.e., the reflected light field component polarized perpendicular to the incident light field. The off-diagonal components are linearly proportional to the magnetization of the sample. Both the diagonal terms r_{pp} and r_{ss} and off-diagonal terms r_{ps} and r_{ps} are complex quantities. The magnitude of the magneto-optical effect is characterized by a complex Kerr angle $\theta + i\varepsilon$, where $\theta = \mathrm{Re}(r_{\mathrm{sp}}/r_{\mathrm{pp}})$ is Kerr rotation and $\varepsilon = \mathrm{Im}(r_{\mathrm{sp}}/r_{\mathrm{pp}})$ Kerr ellipticity.

The reflected beam then passes through a photoelastic modulator with matrix

$$PEM = \begin{bmatrix} e^{-i\varphi_0 \cos \omega t} & 0 \\ 0 & 1 \end{bmatrix},$$

which applies a periodic modulation at a frequency of ω = 50 kHz. Here φ_0 is the amplitude of the retardation. This number can be set by adjusting the modulation amplitude of the photoelastic crystal. The final component is a polarizer placed at angle α with respect to the first polarizer. The electric field at the photodetector is therefore described by

$$\begin{bmatrix} E_{\mathrm{p}} \\ E_{\mathrm{s}} \end{bmatrix} = \begin{bmatrix} \cos^2\alpha & \sin\alpha\cos\alpha \\ \sin\alpha\cos\alpha & \sin^2\alpha \end{bmatrix} \cdot PEM \cdot S \cdot E_{\mathrm{inc}} \,.$$

After matrix multiplication the intensity is calculated from the electric field vector by

$$I = E_p E_{\mathrm{p}}^* + E_{\mathrm{s}} E_{\mathrm{s}}^*,$$

which gives

$$I = E_0^2 \big(r_{\mathrm{pp}} \cos\alpha + r_{\mathrm{sp}}^* \sin\alpha\, e^{-i\varphi_0 \cos\omega t}\big)\big(r_{\mathrm{pp}} \cos\alpha + e^{i\varphi_0 \cos\omega t} r_{\mathrm{sp}} \sin\alpha\big).$$

The intensity of the beam is measured with fast responsive photodetector (DET-100, Hinds Instruments) and a lock-in amplifier is used to filter the signal at the PEM modulation frequency. Further analysis requires decomposition of the exponential terms into harmonics of the retardation frequency by a Jacobi-Anger series [5]. The intensity can then be grouped into DC, 50 kHz (1st harmonic), 100 kHz (2nd harmonic) and higher frequency terms

$$\begin{aligned} I = E_0^2 (r_{\mathrm{pp}}^2 \cos^2\alpha + {r^2}_{\mathrm{sp}} \sin^2\alpha + 2J_0(\varphi_0) r_{\mathrm{pp}} \mathrm{Re}(r_{\mathrm{sp}}) \cos\alpha \sin\alpha + \quad & I_{\mathrm{DC}} \\ 4J_1(\varphi_0) r_{\mathrm{pp}} \mathrm{Im}(r_{\mathrm{sp}}) \cos\alpha \sin\alpha \cos\omega t + \quad & I_{50\,\mathrm{kHz}} \\ 4J_2(\varphi_0) r_{\mathrm{pp}} \mathrm{Re}(r_{\mathrm{sp}}) \cos\alpha \sin\alpha \cos 2\omega t) + \quad & I_{100\,\mathrm{kHz}} \\ \text{higher order terms} \quad & \end{aligned},$$

where $J_i(\varphi_0)$ are the Bessel functions of first kind. The last term of the DC signal can be eliminated by setting J_0 to zero at $\varphi_0 = 0.383 \cdot 2\pi$. Furthermore, the term proportional to r_{sp}^2 is very small and can be neglected. The DC signal and the first two harmonics are read by a lock-in amplifier. The Kerr rotation θ and ellipticity ε are extracted from these signal using

$$\theta = \mathrm{Re}\left({r_{\mathrm{sp}}}/{r_{\mathrm{pp}}} \right) = \frac{I_{100\,\mathrm{kHz}}}{S_{\mathrm{DC}}} \frac{1}{4J_2(\varphi_0)\tan(\alpha)},$$

$$\varepsilon = \mathrm{Im}\left({r_{\mathrm{sp}}}/{r_{\mathrm{pp}}} \right) = \frac{I_{50\,\mathrm{kHz}}}{S_{\mathrm{DC}}} \frac{1}{4J_1(\varphi_0)\tan(\alpha)}\,.$$

In the magneto-optical Kerr spectrometer, an electromagnet is used to orient the magnetization of the Ni nanoparticles perpendicular to the sample plane. Full hysteresis loops of the polar Kerr rotation and ellipticity are measured at each wavelength using magnetic fields up to ± 400 mT. From these

measurements, spectra of ε and $\theta$are extracted by averaging of the saturated magneto-optical Kerr responses (Supplementary Figure 9).

Supplementary References

[1] A Palik, E. Handbook of Optical Constants of Solids I – III (Academic Press, London, 1998).

[2] Krinchik, G. S. & Artemjev, V. A. Magnetooptic Properties of Nickel, Iron, and Cobalt, *J. Appl. Phys.,* **39**, 1276–1278 , (1968).

[3] Maccaferri, N., González-Díaz, J. B., Bonetti, S., Berger, A., Kataja, M., van Dijken, S. Nogués, J., Bonanni, V., Pirzadeh, Z., Dmitriev, A., Åkerman, J., & Vavassori, P. Polarizability and magnetoplasmonic properties of magnetic general nanoellipsoids. *Opt. Express* **21,** 9875-9889 (2013).

[4] Maccaferri, N., Berger, A., Bonetti, S., Bonanni, V., Kataja, M., Qin, Q.H., van Dijken, S., Pirzadeh, Z., Dmitriev, A., Nogues, J., Akerman, J. & Vavassori, P. Tuning the magneto-optical response of nanosize ferromagnetic Ni disks using the phase of localized plasmons. *Phys. Rev. Lett.* **111,** 167401 (2013).

[5] Cuyt, A., Petersen, V., Verdonk, B., Waadeland, H. and Jones, W. B., *Handbook of continued fractions for special functions* p. 344, Springer, (2008).
